# Molecular Analysis of Elements of Melanoma Insensitivity to TCR-Engineered Adoptive Cell Therapy

**DOI:** 10.3390/ijms222111726

**Published:** 2021-10-29

**Authors:** Ali R. Jazirehi

**Affiliations:** 1 Department of Biological Sciences, College of Natural and Social Sciences, California State University, Los Angeles (CSULA), 5151 State University Drive, Los Angeles, CA 90032, USA; Ajazire@calstatela.edu; 2 Department of Life Sciences, Los Angeles City College (LACC), 855 N. Vermont Ave., Los Angeles, CA 90029, USA

**Keywords:** apoptosis, signal transduction, molecular targeted therapy, T cell receptor, vemurafenib, melanoma, resistance, acquired resistance, check point inhibitors, adoptive cell therapy, chimeric antigen receptor

## Abstract

Metastatic melanoma accounts for the highest number of skin cancer-related deaths. Traditional treatments are ineffective due to their inability to induce tumor regression at a high rate. Newer treatments such as immune checkpoint inhibitors (ICI), targeted therapy (BRAFi and MEKi), and T cell receptor (TCR)-engineered T cells aim to increase the ability of the host immune system to recognize and eradicate tumors. ICIs inhibit negative regulatory mechanisms and boost the antitumor activity of the host’s immune system, while targeted therapy directed against aberrant signaling molecules (BRAF and MEK) will block the uncontrolled proliferation and expansion of melanomas. The basis of the TCR-engineered T cell strategy is to transduce host T cells with antigen-specific TCRα/β chains to produce high-affinity T cells for tumor-associated antigens. TCR-transgenic T cells are expanded and activated ex vivo and reinfused into patients to increase the targeting of cancer cells. While these treatments have had varyingly favorable results, their efficacy is limited due to inherent or acquired resistance. Various mechanisms explain melanoma immune-resistance, including the loss or downregulation of the MCH/peptide complex, aberrant activity of signaling pathways, and altered dynamics of apoptotic machinery. Collectively, these mechanisms confer melanoma resistance to apoptotic stimuli delivered by T cells despite a fully functional and effective antitumor immune response. Identification of biomarkers, combination treatment, and the use of CAR T cells are among the approaches that can potentially circumvent melanoma’s resistance to immunotherapy.

## 1. Introduction

Melanoma is responsible for nearly 92% of skin cancer-related diagnoses, as well as 60% of skin cancer-related deaths in the United States alone, with no sign of slowing as the diagnosis of melanoma is increasing at a rate greater than that of other cancers [1]. Accumulating mutations in melanin-producing cells or melanocytes causes the rapid expansion and proliferation of a malignant tumor that either remains localized or disseminates and metastasizes throughout the body. Metastatic cancers, such as melanoma, remain incurable for nearly all patients; this is primarily due to the ability of cancer cells to accumulate various mutations, remain unresponsive to chemotherapy, and evade host immune responses [2]. With melanoma cases rising exponentially in recent years, it is crucial to discuss the role of ultraviolet (UV) light (a mutagen), which is a leading stimulus in melanoma development. Long-term exposure to UV light causes melanocytes to accumulate multiple somatic mutations [3]. Due to the ability of mutated melanocytes to proliferate at an uncontrollable rate and metastasize to various anatomical locations within the body, while remaining unresponsive to chemotherapy, finding a cure for melanoma remains a challenging task. Consequently, melanoma accounts for the highest number of skin cancer-related deaths. This review provides a broad overview of traditional immunotherapeutic approaches, more recent monoclonal antibody (mAb)-mediated targeting of immune checkpoints (PD-1/PD-L1, CTLA-4), and molecular targeting of aberrant signaling molecules (BRAF and MEK), using specific inhibitors for the treatment of melanoma. These serve as a prelude to the focus of this review article, which is the molecular basis for using T cell receptor (TCR)-engineered T cells in the treatment of melanoma and potential mechanisms of resistance. Lastly, we provide potential alternative approaches for the treatment of metastatic melanoma [3].

## 2. Traditional Immunotherapeutic Modalities for the Treatment of Metastatic Melanoma

Traditional treatments used to fight melanoma include surgery, interferon-alpha 2b, (IFN-α2b), high-dose interleukin-2 (IL-2), and tumor-infiltrating lymphocytes (TIL). Surgery is typically the immediate option for patients diagnosed with melanoma that has not reached the metastatic stage. Being able to excise melanoma without the need for other treatments has made surgery the primary treatment option for this cancer, but it is ruled as an ineffective treatment when patients have reached the advanced stages of the disease. Since surgery is unable to treat melanoma patients at advanced stages, specifically metastatic melanoma (mM), new treatments are being developed and assessed in clinical trials.

The cytokine interferon (IFN) alpha 2b (IFN-α2b) possesses antitumor activities and was used as an immunotherapy regimen for the treatment of melanoma. IFN-α2b received FDA approval for the treatment of melanoma patients who have low-volume disease in their soft tissues [4]. The antitumor effects of IFN-α2b are mediated via caspase-independent apoptosis induction in melanomas [5]. Although IFN-α2b causes a wide range of undesired side effects in stage IV melanoma patients including fatigue, fever, and psycho-cognitive impairment [6], it was considered the first agent to confer significant survival benefits (average survival rate: 3.8 years) in clinical investigations [7].

Interleukin-2 (IL-2) is a T cell growth factor that also inhibits tumor growth [8]. IL-2 was one of the earliest molecules to activate T cells in order to invoke a robust immune response. Since being introduced as a treatment option, IL-2 has led to remission in patients with mM [9]. IL-2 was one of two treatments for mM approved by the FDA; however, clinical trials proved that IL-2 could only induce response rates in 10–20% of patients, ultimately leading to IL-2 being disregarded as a potential cure [10]. Although IL-2 was deemed ineffective when used independently, it has a significant role in new immunotherapeutic options in conjunction with several treatments. One of the most important roles IL-2 played in treating mM was shifting attention towards T cells and their ability to be used in immunity-boosting therapies. 

Similar to IL-2, tumor-infiltrating lymphocytes (TIL) shifted attention towards immune system-boosting treatments through the utilization of T cells. The basis for TIL involves isolating T cells that have infiltrated tumor sites and the reinfusion of these naturally occurring T cells into the host to combat cancer cells. TIL therapy consists of three steps—resection, expansion, and reinsertion—in an attempt to invoke robust antitumor immune responses in patients. The first step requires surgical removal of the melanoma and cutting it into small fragments, around 1 mm in size [11]. The identification of activated T cells follows. The process of isolating activated T cells limits the success of TIL due to the difficulty of precisely finding activated T cells in patients diagnosed with mM. Patients diagnosed with the late stages of melanoma have relatively low numbers of activated T cells due to the inherent immunosuppressive properties of the tumor microenvironment. Nevertheless, once this step is completed, TILs will be expanded. T cell expansion occurs over a 28-day period; this timeframe assures adequate activation of T cells; newly expanded T cells will be cryopreserved, followed by reinfusion into the patient [11]. The T cell growth factor, IL-2, is typically used to expand TIL cultures. Activated and expanded TIL cultures will be reinfused back into patients. However, prior to reinfusion, patients receive a lymphodepleting regimen in order to prepare the microenvironment for reinfused TILs. Lymphodepletion increases objective tumor regression in nearly 50–70% of patients, even leading to complete regression in some patients [12]. Although TIL therapy has demonstrated promising results and can be used in treating other cancers such as renal carcinoma, it has been ruled as ineffective as TILs are typically being generated from half of all collected samples [13]. In addition, TIL requires patients to have T cells that are already activated and have penetrated the tumor microenvironment. These difficulties have led to an undeniable need for new therapies to treat mM.

## 3. New Treatment Modalities: Targeted Therapies

### 3.1. Programmed Cell Death-1/Programmed Cell Death-Ligand 1 (PD-1/PD-L1) Immune Checkpoint Blockade

Immune checkpoint inhibitors (ICI) have become a new class of standard treatments that have the potential to cure mM. Programmed Cell Death-1 (PD-1) inhibitors, cytotoxic T lymphocyte antigen-4 (CTLA-4) blockade, and BRAF inhibitors are examples of ICIs used to treat mM, and will be discussed in the following sections. 

**Figure 1:**
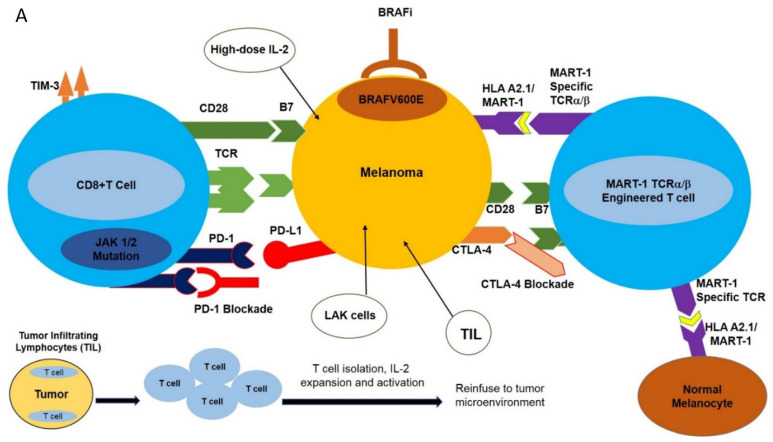

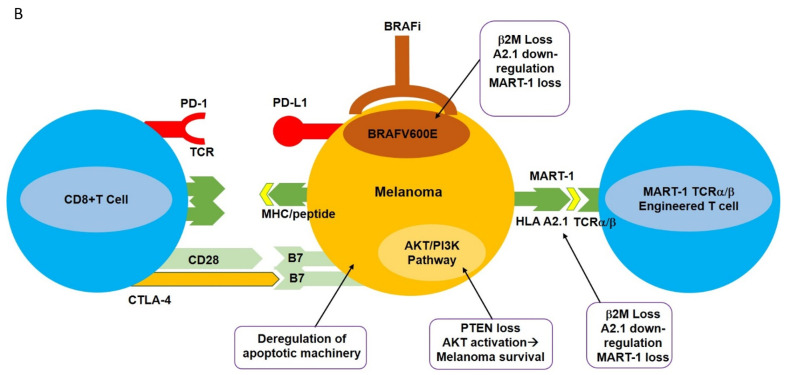
Potential immune-based treatment options for the treatment of metastatic melanoma. (**A**) Various immune-based treatment options are available to treat patients harboring metastatic melanoma, including high-dose IL-2, tumor-infiltrating lymphocytes (TIL), lymphokine-activated killer cells (LAK), and TCR-transgenic T cells directed against specific melanoma-associated antigens such as MART-1. More recently, immune checkpoint blockade using antagonistic monoclonal antibodies directed against PD-1 and CTLA-4, as well as specific molecular targeting of BRAF^V600E^, have been employed. These strategies have had varying degrees of success in the treatment of metastatic melanoma. (**B**) Potential mechanisms of melanoma resistance to T cell-based therapy. Various inherent (primary) or acquired (secondary) mechanisms have been implicated in the resistance of melanoma to T cell-based therapies. These resistance mechanisms may include deregulation of apoptotic machinery, favoring an anti-apoptotic phenotype to melanomas, loss of PTEN, and constitutive activation of the AKT signaling transduction pathway, leading to melanoma survival. Loss of the β2M component HAL A2.1, downregulation (internalization) or shedding of the MHC complex, or loss of TAA (such as MART-1) will render melanomas unrecognized by T cells. For additional information, refer to the text.

Cancer cells promote the production of Programmed Death Ligand-1 (PD-L1), which has the innate ability to bind to PD-1, which is expressed on CD8 T cells [14]. Upon PD-1 binding to PD-L1, the host’s immune system is disabled from detecting cancer cells. PD-L1 can bind to CD8^+^ T cells due to its natural expression in the tumor microenvironment. PD-L1 inhibits TCR signals, which disables the T cells’ ability to recognize and ultimately destroy antigens [14]. Due to the inhibitory effects of PD-1 and PD-L1 on T cells’ ability to combat antigens, melanoma is able to metastasize, or further expand. Treatments that inhibit the binding of PD-L1 to PD-1 are an option for patients with mM. Drugs that inhibit PD-1 include nivolumab and pembrolizumab. The great success of these treatments led to the FDA approval of both of these monoclonal antibodies (nivolumab and pembrolizumab) for the treatment of mM, among other malignancies [9]. The success of PD-1 inhibition stems from its ability to bind to CD8^+^ T cells. In a clinical trial reviewing the mechanisms associated with the benefits of PD-1 inhibition, CD8^+^ T cells must actively express PD-1 prior to PD-1 blockade in order to achieve antitumor activity [14]. After inhibiting PD-1, cancer cells will not be able to go unnoticed, and T cells will be able to identify and attack the cancer cells. Altogether, therapies focused on inhibiting the PD-1 receptor have presented clinical responses in many patients diagnosed with different cancers, including mM (Tumeh et al., 2014). The success of PD-1 inhibition will likely continue to rise if paired with other immunotherapeutic treatments (Figure 1A).

### 3.2. Cytotoxic T-Lymphocyte-Associated Protein (CTLA-4, CD152) Immune Checkpoint Blockade

While PD-1/PD-L1 blockade treatment has shown immense success, CTLA-4 blockade has also displayed an ability to promote the response of the host immune system to cancer cells. The T cell surface receptor CTLA-4 (CD152) has a similar function to that of PD-1/PD-L1: it is an immune checkpoint regulator that is constitutively expressed on regulatory T cells and competes with CD28 in binding to B7.1 and B7.2. Upon binding to B7.1 and B7.2, CTLA-4 downregulates T cell functionality. Upregulation of CTLA-4 in conventional T cells is only observed upon activation, which is frequently seen during malignancies (Figure 1A).

In a cancerous environment, CTLA-4 competes with CD28 protein to bind with B7. The binding of CTLA-4 and B7 leads to T cell production being inhibited, as opposed to the promotion of immune responses that occurs when CD28 binds with B7. CTLA-4 blockade promotes CD28′s ability to freely bind with B7, leading to T cells’ activation and ultimately promoting the destruction of cancer cells. The regression of melanoma experienced when CTLA-4 blockades are used is influenced by the neoepitopes formed when the blockade begins to inhibit CTLA-4. Clinical trials showed that the neoepitopes formed when CTLA-4 blockade is used as a treatment resemble those that T cells naturally identify [15]. The requirements for CTLA-4 to be more effective include the depletion of helper T cells and regulatory T cells [15]. These prerequisites can be met using a lymphodepletion regimen, which was mentioned previously and will be further elaborated upon in a later section. Overall, CTLA-4 blockade treatments, such as ipilimumab, have displayed significant success in treating patients with mM [16]. With remission occurring in nearly 20% of patients, CTLA-4 should be combined with other immunotherapeutic treatments to further increase the efficacy.

## 4. Combination of Immune Checkpoint Inhibitors for the Treatment of Metastatic Melanoma Patients: Summary of Clinical Data

Compared to glycoprotein 100 (gp100), monotherapy with anti-CTLA-4 mAb Ipilimumab (3 mg/kg body weight) could improve overall survival in a phase 3 clinical trial of metastatic melanoma patients who received previous treatment. Investigators further conducted a phase 3 study of ipilimumab (10 mg per kilogram) plus dacarbazine compared to dacarbazine plus placebo in 502 patients with previously untreated metastatic melanoma. The chemotherapy drug dacarbazine (DTIC, also known as imidazole carboxamide) is used in the treatment of melanoma as well as Hodgkin’s lymphoma patients [17]. It was concluded that the overall survival was significantly longer in patients receiving ipilimumab + dacarbazine (9.1 months) compared to those receiving dacarbazine + placebo (11.2 months). The survival rates at 1, 2, and 3 years were higher in the ipilimumab–dacarbazine group. Although drug-related deaths and gastrointestinal perforations did not occur in the ipilimumab–dacarbazine group, grade 3 or 4 adverse events were higher (56.3%) in the combination group (ipilimumab + dacarbazine) compared to dacarbazine + placebo (27.5%)-treated patients [18].

The clinical efficacy and superiority of two immune checkpoint inhibitors, namely the PD-1 pathway blocking mAb (Pembrolizumab or KEYTRUDA,) and anti-CTLA-4 mAb (Ipilimumab or Yervoy), were compared in a randomized, controlled, phase 3 study. Compared to ipilimumab, pembrolizumab significantly prolonged progression-free overall survival events, and reduced the relative risk of death in patients with advanced melanoma. The safety profile of pembrolizumab (KEYTRUDA) was consistent with previous studies; no unexpected safety concerns and fewer grade 3–5 treatment-related adverse events were observed [17].

The human PD-1 blocking mAb Nivolumab blocks the interaction between the PD-1 receptor found on T cells and its ligands PD-L1 and PD-L2, allowing for T cell proliferation and cytokine production. In a phase 3 clinical trial of ipilimumab-refractory metastatic melanoma patients, nivolumab induced higher objective response rates compared to chemotherapy. Investigators conducted a phase 3 controlled study to test the efficacy of nivolumab compared to dacarbazine in 418 previously untreated patients with advanced melanoma without BRAF mutations. The primary end point of this study was the overall survival of patients. The results showed that, at one year, the overall survival rate was higher in the nivolumab group compared with the dacarbazine group (72.9% vs. 42.1%). The median progression-free survival was better in the nivolumab group than in the dacarbazine group (5.1 months vs. 2.2. months). Similarly, the objective response rate was significantly higher in the nivolumab group compared with the dacarbazine group (40% vs. 13.9%). Lastly, drug-related adverse events of grade 3 or 4 were lower in the nivolumab group compared to the dacarbazine group (11.7% vs. 17.6%). Overall, the authors concluded that nivolumab, compared with dacarbazine, is associated with significant improvements in overall survival and progression-free survival, in previously untreated metastatic melanoma patients without a BRAF mutation [19].

Eligible patients with unresectable or metastatic melanoma who had progressed after ipilimumab, or ipilimumab and a BRAF inhibitor were recruited in a multicenter, randomized, controlled, open-label, phase 3 trial, to test the efficacy and safety of nivolumab compared with the investigator’s choice of chemotherapy (ICC) as a second-line or later treatment [20]. Patients were randomly allocated to nivolumab (*n* = 272) or ICC (*n* = 133). Objective response rates were significantly higher in the nivolumab group than in the ICC group. Grade 3–4 adverse events attributed to nivolumab included increased lipase, increased alanine aminotransferase, anemia, and fatigue; those for ICC included neutropenia, thrombocytopenia, and anemia. Overall, serious grade 3–4 drug-related adverse events occurred in 5% of nivolumab-treated patients and 9% of patients in the ICC group, and no treatment-related fatalities were reported. These investigators concluded that nivolumab can lead to a higher proportion of patients achieving objective responses and fewer toxic effects compared with the alternative available chemotherapy regimens for patients with advanced melanoma who have progressed after ipilimumab or ipilimumab and a BRAF inhibitor [20].

## 5. Targeted Therapy of Aberrant Signaling Molecules for the Treatment of Metastatic Melanoma Patients 

The MAPK (Mitogen Activated Protein Kinase) signal transduction pathway controls the proliferation, differentiation, and survival of the cells. The ERK1/2 (extracellular signal-regulated kinase) signaling pathway is the major MAPK signaling pathway, mainly involved in the proliferation and survival of tumor cells, leading to their growth [21]. The components of the ERK1/2 pathway include the GTPase RAS, which, upon activation, activates the RAF molecules (e.g., ARAF, BRAF, CRAF) and subsequently phosphorylates MEK and ERK kinases. BRAF is principally activated by RAS and, once mutated, acts as a monomer-independent stimulus (Figure 1A) [22,23,24,25,26]. 

Approximately 60% of melanoma patients harbor various BRAF mutations; 90% of these mutations are substitutions of glutamic acid for valine at position 600; BRAF^V600E^, leading to constitutive MAPK activation, tumor proliferation, and a significant (500X) increase in BRAF kinase activity compared to wild-type BRAF [27,28]. The oral serine-threonine kinase inhibitor called vemurafenib (PLX4032, RG7204) is a BRAF^V600E^-specific inhibitor that inhibits the MAPK pathway and MEK and ERK phosphorylation, induces cell cycle arrest, and activates apoptotic pathways in BRAF ^V600E^-mutated cells [26]. 

Vemurafenib has improved the survival rate in patients with metastatic melanoma harboring BRAF^V600E^ mutations. After successful in vitro and in vivo experiments, vemurafenib is considered a promising drug for use against melanoma (discussed below). However, melanomas treated with vemurafenib will eventually adopt various resistance mechanisms that progress over time, leading to eventual relapse [27,28,29]. 

The MEK inhibitor AZD6244 efficiently inhibits the MAPK pathway; however, its benefits are often offset as it impairs T cell function [30]. Another MEK inhibitor called PD0325901 produced a significant decrease in ERK phosphorylation and led to disease stabilization in phase I clinical trials (Figure 1A) [31,32].

## 6. Treatment of Metastatic Melanoma Patients Harboring BRAF^V600E^: A Summary of the Clinical Data

Dabrafenib (GSK2118436) is another selective ATP-competitive BRAF ^V600E^ inhibitor. Like vemurafenib, dabrafenib has selectivity towards the mutant BRAF but not the wild type [33]. In contrast to MEK inhibitors, dabrafenib (GSK2118436) combined with ACT (adoptive cell transfer) did not suppress the functionality of patient lymphocytes (Figure 1A).

Clinical investigation of patients with V600E, V600K, and V600D mutations and patients with untreated brain metastasis had an average progression-free survival of 8.3 months upon dabrafernib (GSK2118436) treatment. Dabrafernib produced similar results to vemurafenib in dose-escalating studies. Melanoma patients harboring V600E/K mutations showed a response rate of about 60% in phase I and 59% in phase II trials [34]. 

Due to promising preclinical data, vemurafenib’s effects were further evaluated in metastatic melanoma patients harboring BRAF^V600E^ mutations. Successful phase I [35] and II trials [36] led to a comparison of vemurafenib and dacarbazine in previously untreated BRAF^V600E^ expressing metastatic melanoma patients in a phase III trial (BRIM-3). The median survival with vemurafenib was 16 months compared to less than 10 months for dacarbazine. Response rates for vemurafenib and dacarbazine were 48% and 5%, respectively. The vemurafenib group exhibited a reduction of 63% in the risk of death and 74% in the risk of death or disease progression, relative to dacarbazine. Due to these benefits, the FDA approved vemurafenib on 17 August 2011 [37]. Considering all phase studies, vemurafenib is a drug with augmented rates of overall survival in melanoma patients with a BRAF V600E mutation.

RAF265 is another BRAF ^V600E^ inhibitor that reduces tumor growth and induces tumor regression. The response rates were 16% in patients with a BRAF mutation and 13% in patients with WT BRAF. The results are moderate relative to those of other BRAF inhibitors [38]. The drug might cause more toxicity rather than increasing any feasible antitumor activity. Recently, the effect of intermittent dosing of dabrafenib (BRAF^V600E^ inhibitor) and trametinib (MEK inhibitor) in patients with metastatic and unresectable BRAF^V600^ melanoma was evaluated in a randomized, open-label, phase 2 clinical trial. Intermittent dosing did not improve progression-free survival [39]. However, the results of a recent clinical trial using a combination of BRAF inhibitor and HSP (heat shock protein) inhibitor (XL888) showed that 20% (3/15) of metastatic melanoma patients achieved complete remission, while 80% (12/15) experienced partial responses, suggesting the potential benefit of the inclusion of HSP inhibitors in clinical trials to overcome BRAFi resistance [40].

## 7. Recent T Cell-Based Immune Therapies for the Treatment of Metastatic Melanoma Patients

### 7.1. Molecular Basis for Using TCR-Engineered T Cells

Monoclonal antibody-mediated blockade of immune checkpoints such as PD-1/PD-L1 and CTLA-4, as well as targeted inhibition of BRAF^V600E^, have had profound results in various clinical trials. However, the clinical utilization of these modalities is hampered by the development of resistance as well as limited response rates. This has spurred the design of new strategies with high selectivity towards melanomas including adoptive cell transfer (ACT). This new immunotherapy-based treatment involves ex vivo expansion and activation of HLA A2.1/MART-1-specific T cell receptor (TCR) engineered T cells and reinfusion of these newly expanded high-affinity MART-1-specific T cells into patients with metastatic melanoma [41]. Heterodimeric T cell receptors (TCR) consist of two transmembrane polypeptide chains, one alpha chain, and one beta chain. Each chain devotes a portion of itself for stable attachment on the T cell surface, and a variable region responsible for binding to the Major Histocompatibility Complex (MHC) [1]. TCRα/β-engineering gives T cells high specificity and increased affinity for the MART-1 melanoma-specific antigen expressed in the context of HLA A2.1 (Figure 1A) [42]. 

While traditional ACT has shown promise, modifications have been made to the protocol to increase the efficacy of treatment. These fine-tunings include the pre-infusion of a lymphodepleting regimen, and postinfusion use of high-dose IL-2. Lymphodepletion prior to the infusion of T cells has been proven to promote the antitumor response. This is possibly because of its ability to promote an environment that is cleared of potentially inhibiting molecules [43]. Lymphodepletion has been proven to promote the recruitment of homeostatic cytokines IL-15 and IL-17 to the tumor milieu in clinical trials [44], and also provides the physical space for TCR transgenic T cells. IL-2 has the innate ability to promote T cell growth; therefore, its postinfusion inclusion in clinical settings of TCR-engineered T cell therapy of melanoma ensures the sustained expansion and activation of infused MART-1 TCR T cells.

### 7.2. Clinical Trials Involving TCR Immunotherapy: Molecular Aspects 

Several clinical trials have been conducted to identify melanoma-specific antigens that can be targeted by TCR-engineered T cells. Results of an early ACT clinical trial using melanocyte differentiation antigen-1 (MART-1)-specific TCRα/β deemed it not efficacious as only two of the 15 patients exhibited partial and nondurable responses to treatment. These results were significantly lower than those observed by TIL therapy; however, TCR immunotherapy has been favored due to its broader application in wider patient demographics [45]. Additionally, this pioneering trial showed that TCR immunotherapy had tremendous potential compared to contemporary treatments [46].

In a separate clinical trial, tyrosinase-specific TCR was used to treat patients with melanoma. The rationale for using tyrosinase TCR was that it could potentially promote the functionality of CD4 and CD8 T cells [45]. Ex vivo activation of tyrosinase-specific TCR T cells was accomplished in the presence of CD34 cells, followed by IL-2 and IL-15-mediated expansion. Following lymphodepletion regimen, fully activated tyrosinase-TCR T cells are reinfused into patients along with IL-2 [45]. Similar to the MART-1-specific TCR T cell trials, the results of this trial ultimately led to tyrosinase-specific TCR being declared ineffective: two of the three patients died, with the living patient experiencing a minimal improvement. 

Overall, TCR-engineered melanoma immunotherapy has led to varying responses, ranging from being ineffective to complete remission. TCR-engineered ACT will thus continue to play a significant role in cancer immunotherapy. Due to its specificity and high affinity for tumor-associated antigens, combined with its potential efficacy, TCR-engineered T cell ACT remains a preferred treatment option [47]. However, TCR-engineered T cell therapy protocols require further optimization to increase the sustainability and efficacy of genetically modified T cells in the treatment of metastatic cancers including melanoma. Combination of TCR-engineered T cell ACT with ICIs to increase treatment efficacy may be a plausible area of future investigation [48].

### 7.3. Molecular Analysis of the Failure of TCR-Engineered T Cell Therapy: Adverse Effects

While TCR-engineered T cell-based immunotherapy has the potential to treat mM, it also has the potential to induce undesired toxic side effects in the host. These adverse effects can contribute to the failure of immunotherapy. The most common adverse events that challenge, and in some instances, cause the failure of T cell therapy include cytokine release syndrome (CRS) and autoimmune responses.

#### 7.3.1. Cytokine Release Syndrome (CRS)

Cytokine release syndrome (CRS) occurs after the infusion of activated transgenic T cells and is exacerbated by the introduction of high doses of IL-2. CRS is due to the release of large quantities of cytokines and is the result of a robust increase in the activity of the host immune system to a level greater than that of the normal homeostatic state [2]. The most challenging aspect of CRS is its tendency to induce higher toxicity in patients who receive larger numbers of TCR-transgenic T cells. Typically, patients with advanced stages of mM require stronger treatments, making the development of CRS inevitable in many cases. Patients typically experience side effects including headaches, seizures, loss of memory, and loss of consciousness. While these side effects seem minor, more severe toxicities such as cardiac dysfunction, lung inflammation, severe neurological damage, and severe blood clots may occur [2]. CRS can potentially lead to patients’ demise. The most common treatment for CRS is the IL-6 inhibitor tocilizumab [2]. 

#### 7.3.2. Autoimmune Responses

Aside from CRS, autoimmune responses pose a tremendous challenge for patients receiving ACT treatment with TCR-engineered T cells. Autoimmune responses are the result of “On Target, Off Tumor” phenomena. This “On Target, Off Tumor” response results from newly infused T cells attacking the correct antigens, but in the wrong locations. Tumor-associated antigens (TAA) are expressed not only on tumors but also on naturally occurring, nonmutated cells [2]. There is currently no strategy that can ensure newly infused TCR-engineered T cells will only target mutated/overexpressed tumor-associated antigens, as was demonstrated for MART-1-specific T cells. The newly infused TCR-engineered T cells were expected to attack the MART-1/HLA A2.1 complex on melanomas. However, it was later discovered that MART-1 antigens are not only expressed on melanomas, but also on naturally occurring melanocytes [2]. Patients who received this treatment typically experienced vitiligo in one or more locations. Similarly, in the context of clinical utilization of MAGE-A3-specific T cells, at the time of treatment it was unclear whether MAGE-A3 was also expressed in the human brain. Postclinical trial results included five patients showing tumor regression, three patients experiencing neurotoxic effects, and two patients who had died [46]. These clinical results were due to the newly infused MAGE-A3 TCR transgenic T cells attacking the MAGE-A3 antigens expressed in the brain.

## 8. Molecular Mechanism of Resistance to TCR-transgenic T-Cell Adoptive Cell Transfer Immunotherapy 

The treatment modalities described above induce tumor regression in a subset of patients with mM; however, metastatic cancers remain incurable for the vast majority of patients. This is mainly due to the inherent ability of metastatic cancer cells to escape various treatments including ACT as well as targeted therapies [49]. Patients who receive ICI treatments or TCR immunotherapy may exhibit primary (inherent) or acquired (secondary) resistance to these treatment options. Primary resistance refers to the innate presence of molecules being present when treatment begins. Adoptive or acquired immune resistance is primarily the process in which activated and functionally competent TCR transgenic T cells successfully attack cancer cells, but cancer cells acquire additional mutations to withstand T cell attacks [50]. Detailed cellular and molecular analysis of patient samples who experienced exceptional responses to immunotherapy revealed that certain criteria must be met to ensure patients do not experience primary resistance, or develop acquired resistance [49]. Essentially, these factors include the possession of T cells with an inherent ability to specifically recognize and have high affinity for binding to tumor-associated antigens, the ability to proliferate in response to appropriate stimuli (IL-2, CD3 cross-linking, CD28, etc.), and the ability to maintain their functionality once they arrive in the tumor microenvironment [51]. The mechanisms by which TCR transgenic T cells will be rendered ineffective pre- and postinfusion will be discussed in the following sections (Figure 1A).

### 8.1. Primary Resistance to Immunotherapy 

#### 8.1.1. Defects in the Major Histocompatibility Complex (MHC)

The MHC is a major factor determining the outcome of ACT therapy. In early trials, mutations in MHC were the major cause of ACT immunotherapy failure [52]. The MHC plays a key role in immune responses; tumor-associated antigens (TAA) are being presented in the context of MHC and recognized by the T cell receptor as a prerequisite for T cell activation [49]. However, MHC can mutate in cancer cells, which will ultimately prevent TCR from identifying and binding to TAAs. MHC can also be downregulated or shed from cancer cells’ surface. These events will also contribute to tumor cell evasion from immune attack. The MHC of patients unresponsive to immunotherapy were beta-2 microglobulin (β2M)-deficient [52]. The ability of cancer cells to downregulate β2M allows them to proliferate rapidly and evade detection by T cells. As a result, patients harboring tumor cells with low/undetectable β2M will likely not respond to TCR immunotherapy. This form of resistance may be overcome with the introduction of a new ACT-based treatment, which will be discussed later (Figure 1B). 

#### 8.1.2. Aberrant PI3K/AKT(PKB) Signaling Pathway and Loss of Phosphatase and Tensin Homolog Gene (PTEN)

While MHC defects play a role in primary resistance, another major factor influencing the outcome of TCR-transgenic T cell ACT is the aberrant activation of various signal transduction pathways, which ultimately confer an apoptosis-resistance phenotype to tumor cells. The phosphatidylinositol 3-kinase (PI3K) pathway, also known as AKT (protein kinase B/PKB) plays a significant role in tumor cell expansion and survival, and thus has a tremendous effect on how patients respond to immunotherapy [53]. There is an inverse relationship between activation of the PI3K pathway and the intracellular levels of the tumor suppressor phosphatase and tensin homolog gene (PTEN). Thus, loss or mutations of PTEN paired with constitutive activation of the PI3K pathway, as frequently observed in the vast majority of melanoma patients, will dictate how patients will eventually respond to ICI therapy. High response rates were observed in a study of 39 patients with mM who received PD-1 blocking mAb; the response rate directly correlated with the levels of PTEN. Patients with high PTEN expression levels showed higher rates of tumor regression compared to those with low PTEN levels [53]. Activation of the AKT signaling pathway will induce the expression of vascular endothelial growth factor (VEGF). VEGF plays a role in the rapid growth of tumor cells and is upregulated when there are low levels of PTEN. Additionally, there is an inverse relationship between PTEN and VEGF expression levels. The main reason for the observed differences in tumor regression is likely due to the expression of vascular endothelial growth factor (VEGF). Therefore, PTEN levels (or mutational status) contribute significantly to the outcome of ACT clinical trials (Figure 1B). 

#### 8.1.3. Mutations in the Janus Kinase (JAK1/2) Signaling Module

Similar to PTEN, signaling molecules in the Janus Kinases (JAK) signal transduction pathway can potentially affect the host’s immune system to induce primary resistance to immunotherapy. Mutations in JAK1/2 can potentially cause the host immune system to remain unresponsive to PD-1 inhibitory treatments [54]. In order to gain a deeper understanding of how JAK1/2 potentially promotes primary resistance, it is important to remember the mode of action of ICI PD-1. Anti PD-1 immunotherapy functions by activating T cells that have been anergized by PD-L1-expressing tumor cells. T cells are rendered inactive upon PD-1 binding to PD-L1 on the tumor cell surface. Mutations in JAK1/2 will hamper the ability of T cells to receive cytokine signaling; thus, they remain inactive, which in turn will allow cancer cells to remain undetected by T cells, leading to primary resistance to immunotherapy. Interferon gamma receptor (IFN-γ) is the primary receptor affected by JAK1/2 mutations. Cell lines with JAK1/2 mutations remained unresponsive to IFN-γ, leading to an increase in PD-L1 expression [54]. IFN-γ is produced by T cells upon binding to cancer cells and promotes antitumor immune responses via a variety of mechanisms, including increased expression of tumor-associated antigens (TAA), recruitment and activation of additional immune cells, and promotion of apoptosis of tumor cells [54]. Notably, JAK1 is implicated in the initiation of signals from IFN-alpha/beta and gamma receptors, while JAK2 signals from IFN-γ receptors [54]. Therefore, patients harboring JAK1/2 mutations will exhibit primary resistance to immunotherapy (Figure 1B). 

### 8.2. Adaptive (Inherent/Primary)/Acquired (Secondary) Resistance to Immunotherapy

#### 8.2.1. Treatment-Induced Hyperprogression and Potentiation of Tumor Cell Resistance to Immunotherapy

Interferons such as IFN-γ significantly increase the antitumor effects of the host immune system during immunotherapy. However, overproduction of interferons may influence the response to immune-based antitumor treatments, such as PD-L1, and indolamine 2, 3 dioxygenase (IDO) upregulation [50]. The observed IDO upregulation allows cancer to thrive in the newly, antitumor-derived environment. A recent study evaluating patients who developed resistance to anti-PD-1 blocking antibodies reported that 12 of 131 patients experienced rapid tumor growth following treatment [55]. The authors concluded that the observed post-treatment hyperprogression of tumors is the result of overproduction of interferons and, ultimately, inhibits antitumor activity. Overall, a cohort of patients acquire resistance to the PD-1 ICI because of the inhibition of T cell function and potentially rapid expansion of tumors. 

#### 8.2.2. Role of T-Cell Immunoglobulin Mucin 3 (TIM-3)

Primary or inherent resistance results in unresponsiveness to initial treatment, which remains a major hurdle in the successful treatment of cancer. However, acquired or adaptive resistance is also experienced by a large number of patients who have initially responded to treatment. Overexpression of T-cell immunoglobulin mucin 3 (TIM-3) has been identified as a potential mechanism influencing the development of acquired resistance. The TIM-3 receptor regulates the function of helper T cells. However, TIM-3 also has the ability to impair the p38MAPK signaling pathway, which regulates cell death [56]. The Tim-3 receptor is more abundant in areas that are experiencing T-cell exhaustion—a phenomenon observed in patients that showed unresponsiveness to PD-1 inhibitory treatment [52]. While TIM-3 itself may not be responsible for influencing acquired resistance, its detection in the tumor microenvironment may aid in identifying why treatment is regressing (Figure 1B).

#### 8.2.3. Role of Apoptosis Machinery in Melanoma Resistance to T Cell-Based Therapies 

Current adoptive cell transfer approaches focus only on lymphocytes and overlook the inherent/acquired resistance of melanomas. Most ACT strategies seek to achieve a robust and long-lived CTL response; however, as evidenced by various studies [57,58], even in the face of highly avid and specific CTLs, a large population of tumor cells do not respond to the apoptosis induced by CTLs, resulting in limited response rates. The emergence of immune-resistant tumor variants (which also exhibit cross-resistance to other modalities) remains a major problem in successful cancer therapy. Most chemotherapeutic agents, as well as CD8^+^ cytotoxic T lymphocytes (CTL), induce apoptotic cell death to eliminate tumor targets. Conversely, malignant cells adopt different approaches to evade or resist apoptosis. For example, inhibitors of apoptosis naturally present in tumor cells (e.g., anti-apoptotic Bcl-2 and IAP family members) guard melanomas from apoptotic death in response to drugs and immune cells [59,60,61]. Diminished expression of pro-apoptotic proteins and death receptors or overexpression of anti-apoptotic Bcl-2 and IAP family members confers resistance to the apoptotic stimuli delivered by CTLs. The other issue concerning melanoma resistance to apoptosis is the failure of the cells to carry out the signaling pathways, ultimately leading to cell death. This may be due to insufficient expression of signaling molecules (death receptors such as Fas, DR4, or DR5), overexpression of protective factors (Bcl-2, Bcl-xL, or Mcl-1), mutations in anti-apoptotic proteins (caspase-9 or Ras), or transcriptional silencing of pro-apoptotic factors (Apaf-1, PTEN, Bax, or Bad). The expression of these apoptosis-resistant gene products is regulated by several signal transduction pathways (e.g., NF-κB or MAPK) that are constitutively activated/deregulated in resistant tumors. Melanomas often arise due to mutations affecting cellular signaling pathways that regulate cell proliferation and survival such as B-RAF, N-RAS, NF-κB, MAPK, and AKT/PKB [60,61]. By identifying the main operating network(s), one can, in principle, design a combination of agents targeting these aberrant signaling molecules. Therefore, drugs or biological response modifiers that adversely modify the dynamics of aberrant signal transduction pathways operative in tumors that are capable of modifying the expression profile of apoptosis-related proteins may be successfully used in combination with cell-based therapy protocols in the clinical treatment of metastatic melanomas. The long-term objective of research should focus on determination of the major cellular signaling pathways regulated by sensitizing agents that avert the resistant phenotype of CTL-refractory melanomas (biomarkers). In this respect, the functional complementation (two signal) model was proposed [59]. According to this model, treatment of melanomas with a nontoxic sensitizing agent alters the expression profile of apoptosis-associated gene products (signal I), removes the inhibitory block in the apoptotic pathway, and, by lowering the apoptosis threshold, sensitizes melanomas to the cytotoxic effects of the second agent (e.g., T cell-based therapies) (signal II).

## 9. Potential Mechanisms for Reversing TCR Immunotherapy Resistance

### 9.1. Paired ICI Treatments (PD-1 Paired with CTLA-4)

The results of clinical studies provide evidence that checkpoint inhibitors, used alone, are insufficient to induce permanent responses in all patients [53]. This led to the hypothesis that combined ICI treatment modalities may produce higher results than independent therapies since each ICI inhibits different molecules. For instance, the ICI CTLA-4 is primarily influential in lymphoid tissue, as opposed to PD-1 ICI, which affects the tumor microenvironment [62]. The combination of CTLA-4 and PD-1 blockade restored IL-2 production and CD8 T cell expansion in a murine model; however, no additional T cell infiltration into the tumor microenvironment was observed. Therefore, it is possible that a combination of various ICI immunotherapies can restore the negative (inhibitory) effects of tumor microenvironment on T cells [63], resulting in more favorable clinical responses. 

### 9.2. Biomarker Discovery 

Melanomas are diagnosed at various stages of the disease and in different anatomical positions, which makes it rather difficult to determine and choose the appropriate treatment options. The identification of biomarkers may present clinicians with the ability to determine which treatments will be successful and which may fail due to the development of resistance. One such biomarker informing clinicians what ICI not to use to treat patients is lymphocyte activation gene 3 (LAG-3), a molecule expressed on the surface of and affecting the functionality of regulatory T cells (Tregs) [64]. The immune inhibitory receptor CTLA-4 also naturally occurs on Tregs [65]. Therefore, the identification of high levels of LAG-3 would alert clinicians that there is likely an upregulation of Tregs, and CTLA-4 blockade immunotherapy could potentially be used. Similarly, the molecule TIM-3 is upregulated both in patients who reject anti PD-1 therapy and in those who have acquired resistance. Thus, the identification of elevated levels of TIM-3 would allow physicians to either shift treatment to another ICI or attempt to use a TIM-3 inhibitory treatment in conjunction with ICI. Treatment with TIM-3 inhibitory antibody led to a survival advantage in a murine model [65]. This not only displays TIM-3’s potential as a biomarker, but also highlights the potential of targeting TIM-3 as a therapeutic option. 

### 9.3. Chimeric Antigen Receptor (CAR) T Cell-Based Immunotherapy for Metastatic Melanoma 

The downregulation or loss of the MHC complex on the tumor cell surface is a major mechanism limiting the efficacy of T cell-based therapies. This problem can potentially be circumvented by using chimeric antigen receptors (CAR)-based T cell therapy. Traditionally, T cell receptors recognize tumor cells via binding to the MHC/peptide complex expressed on the surface of cancer cells; however, the recognition of tumors by CAR T cells is MHC-independent. CARs are genetically engineered to use a single-chain variable fragment (scFv) antigen recognition domain bound to a T cell signaling domain [66]. The outer portion of the CAR contains the scFv, which receives and transmits the signal to the activation domain for optimal T cell activation. CAR T cells are advantageous as they provide the possibility of the addition of costimulatory domains to promote CAR T cell proliferation as well as stable CAR expression on the T cell surface. Several excellent review articles have described CAR T cell-based therapies and various CAR constructs; therefore, in order to avoid redundancy, these issues will not be addressed here. Recent advances in CAR T cell manufacturing and mass production and the inclusion of a lymphodepleting regimen prior to CAR T cell infusion have contributed to the superior results obtained by CAR T cell-based immunotherapies for various cancers [66].

While clinical trials testing the efficacy of CAR T cells in melanoma are limited, CAR T cells have shown high efficacy in treating B cell malignancies. In a clinical trial using CD19-redirected CAR T cells, six of eight patients with hematological malignancies experienced high clinical response rates, with one displaying a complete response [67]. The success of CAR T cells in treating hematological malignancies such as acute lymphoblastic leukemia (ALL) and non-Hodgkin’s lymphoma (NHL) has increased interest in testing CAR T cells to treat metastatic melanoma. As CAR T cells recognize tumor cells in an MHC-independent manner, they have the potential to treat patients displaying primary resistance to TCR immunotherapy. 

## 10. Discussion

Traditional treatment modalities have resulted in transient responses in patients harboring metastatic melanoma. More advanced treatments such as immune checkpoint inhibitors (ICI) have induced tumor regression in a large number of patients. Similarly, TCR-transgenic T cells aim to boost the ability of T cells to recognize, bind to, and induce apoptosis in melanoma cells with higher efficacy. This treatment approach ensures that the majority of T cells in the tumor microenvironment remain active and target cancer cells with high specificity. While early trials of TCR-engineered T cells (e.g., MART-1 TCR, tyrosinase TCR, etc.) have shown various degrees of efficacy in inducing tumor regression in patients with metastatic melanoma, further improvements to TCR constructs or modifications to the treatment protocols are needed to increase the efficacy of TCR-engineered T cells in the treatment of melanoma.

Despite moderate initial response rates, patients treated with ICI and TCR-engineered T cells will eventually relapse due to inherent (primary) and/or development of acquired (secondary) resistance to therapy. These resistance mechanisms include loss/downregulation of MCH complex, aberrant dynamics of signal transduction pathways (e.g., AKT/PKB, p38MAPK, JAK/STAT, PTEN, etc.), dysregulation of apoptotic machinery, etc. These mechanisms confer apoptosis resistance to melanomas despite the presence of a fully functional and effective antitumor immune response.

The development of resistance highlights the urgent need to develop novel treatments and/or modifications to existing protocols to increase the efficacy of antitumor immune responses. One of these approaches is the identification of biomarkers that determine patient sensitivity or resistance to a particular treatment. Another approach is to combine various treatment modalities, as each treatment targets a specific molecule/pathway in the tumor cells and the combination may result in synergistic effects. Lastly, serious considerations should be given to CAR T cell-based treatments based on their great promise in the treatment of other cancers.

## Data Availability

All important data is included in the manuscript.
